# Neuron Circuit Based on a Split-gate Transistor with Nonvolatile Memory for Homeostatic Functions of Biological Neurons

**DOI:** 10.3390/biomimetics9060335

**Published:** 2024-05-31

**Authors:** Hansol Kim, Sung Yun Woo, Hyungjin Kim

**Affiliations:** 1 School of Electronic and Electrical Engineering, Kyungpook National University, Daegu 41566, Republic of Korea; khs8465khs@naver.com; 2 Division of Materials Science and Engineering, Hanyang University, Seoul 04763, Republic of Korea

**Keywords:** neuron circuit, homeostasis functionality, nonvolatile memory, charge trap layer

## Abstract

To mimic the homeostatic functionality of biological neurons, a split-gate field-effect transistor (S-G FET) with a charge trap layer is proposed within a neuron circuit. By adjusting the number of charges trapped in the Si_3_N_4_ layer, the threshold voltage (V_th_) of the S-G FET changes. To prevent degradation of the gate dielectric due to program/erase pulses, the gates for read operation and V_th_ control were separated through the fin structure. A circuit that modulates the width and amplitude of the pulse was constructed to generate a Program/Erase pulse for the S-G FET as the output pulse of the neuron circuit. By adjusting the V_th_ of the neuron circuit, the firing rate can be lowered by increasing the V_th_ of the neuron circuit with a high firing rate. To verify the performance of the neural network based on S-G FET, a simulation of online unsupervised learning and classification in a 2-layer SNN is performed. The results show that the recognition rate was improved by 8% by increasing the threshold of the neuron circuit fired.

## 1. Introduction

**Figure 1:**
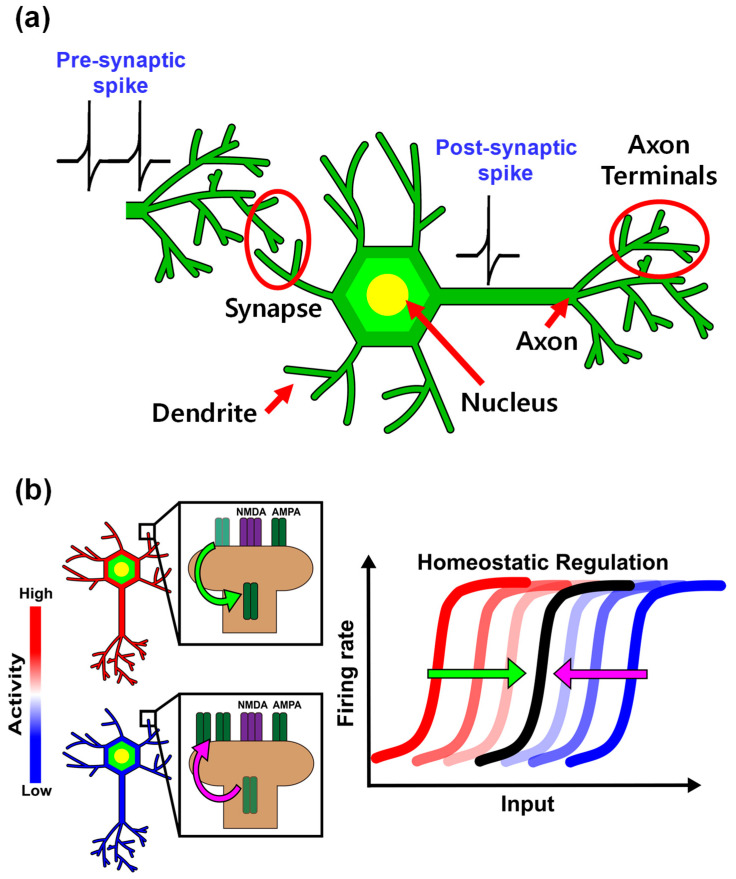
(**a**) Simple diagram of a biological neuron and synapse. The neuron is composed of a nucleus, dendrite, axon, and axon terminals. Synapse is a connection between pre-synaptic neurons and post-synaptic neurons. (**b**) A model of homeostatic regulation which stabilizes firing rate of neurons by synapse scaling.

Recently, artificial neural networks (ANNs), inspired by the brain, have been actively researched and developed [1]. Particular attention has been paid to spiking neural net-works (SNNs) that utilize biological spike timing–dependent plasticity (STDP) rules to function as event-driven systems for unsupervised learning [2,3,4,5,6,7,8,9,10]. Since such SNNs pursue a more bio-plausible direction than other ANNs do, it is necessary to explore the signal transmission mechanisms of biological neurons and synapses to gain a deeper understanding of these SNNs. Figure 1a illustrates the neurons and synapses, and we focus on explaining the membrane potential and neurotransmission of neurons. Neurons maintain the membrane potential by consuming adenosine triphosphate, and the mem-brane potential increases when cations are injected into the membrane due to stimuli pro-vided as dendrites. At this point, if the membrane potential exceeds a specific threshold value, the neuron will be depolarized. Then, the membrane potential reaches the action potential, following which the neuron is repolarized and the membrane potential restored to its initial value. The electrical spikes generated in this process are transmitted to the axon of the neuron in an electrical form and to the post-synaptic neuron in the form of a neurotransmitter through the synapse. However, according to rate-based learning rules such as SRDP and BCM [11,12,13,14], synapses cannot simply transmit signals between two neurons; rather, they can modulate the strength of the transmitted signal by increasing the synaptic efficiency when receiving high-frequency stimulation and reducing it when receiving low-frequency stimulation. These changes in synaptic strength can be explained primarily by activity-dependent plasticity, such as long-term potentiation and long-term depression, and are known to be closely related to the functions per-formed by the brain, such as learning, memory, and cognition. When synaptic strength changes only due to activity, the neural activity can be induced in the direction of runaway excitation or sequences. In response, neurons are known to perform homeostatic regulation to stabilize natural activity and maintain their average firing rate within a narrow range. Figure 1b shows the change of AMPA receptor’s number at the post-synaptic surface induced by neural activity. AMPA receptor’s numbers at the postsynaptic surface are accordingly scaled up- or downwardly in response to activity deprivation or overexcitation, respectively. In this process, the firing rate of neurons is maintained in a narrow range [15,16,17,18].

Recently, research has focused on integrating neuromorphic systems and hard-ware-based neural networks with various emerging memory devices to enable energy-efficient operation of artificial intelligence algorithms [9,10,19,20,21,22,23,24,25,26,27,28,29,30,31,32,33,34,35,36,37,38,39,40,41,42,43]. For instance, on-chip learning can reduce power consumption and compensate for the degradation caused by device variation [44,45,46,47,48,49]. Similar to biological neurons, STDP-based neural networks re-quire a homeostasis functionality that controls the firing rate of the output neurons to achieve more accurate pattern recognition. The homeostasis functionality in biological neurons involves reducing the fire rate of dominant (high firing rate) neurons and increasing that of weak (low firing rate) neurons. Various studies have attempted to implement the homeostasis functionality in hardware and software neural systems [50,51,52,53,54,55,56]. To mimic the homeostasis function, each neuron needs peripheral circuits composed of conventional complementary metal-oxide-semiconductors (CMOSs) and capacitors. However, if all neuron circuits have at least one large capacitor, the hardware footprint of the neural system can become excessive. In addition, when synaptic weights are updated in SNNs, the value controlling the firing frequency of each neuron circuit is also updated and stored in the peripheral circuits. Due to the volatile memory functionality in peripheral circuits, maintaining the values for the homeostasis functionality in the system is impossible without a supply voltage.

In this paper a split-gate field-effect transistor (S-G FET) with a charge-storage layer is proposed to mimic the homeostasis functionality. The S-G FET is used to compare the membrane potential (*V*_mem_) with the threshold voltage (V_th_) for the fire function in the neuron circuit. By using the fin structure in the S-G FET with independent double gates, it was possible to implement a stable operation of neuron circuit by distinguishing between the gate for implementing homeostatic function through P/E and the gate for reading synaptic signals. The V_th_ of each neuron circuit can be updated selectively using the S-G FET, which has two independent gates. The homeostasis functionality can be achieved by controlling the threshold of the neuron circuit using the program/erase operation in the charge-trap layer. Employing a nonvolatile memory functionality for the charge storage layer can maintain the optimized V_th_ of the neuron circuit. A two-layer SNN based on biological STDP learning rules was simulated with the S-G FET to demonstrate the improved pattern-recognition accuracy on MINST datasets.

## 2. Materials and Methods

**Figure 2:**
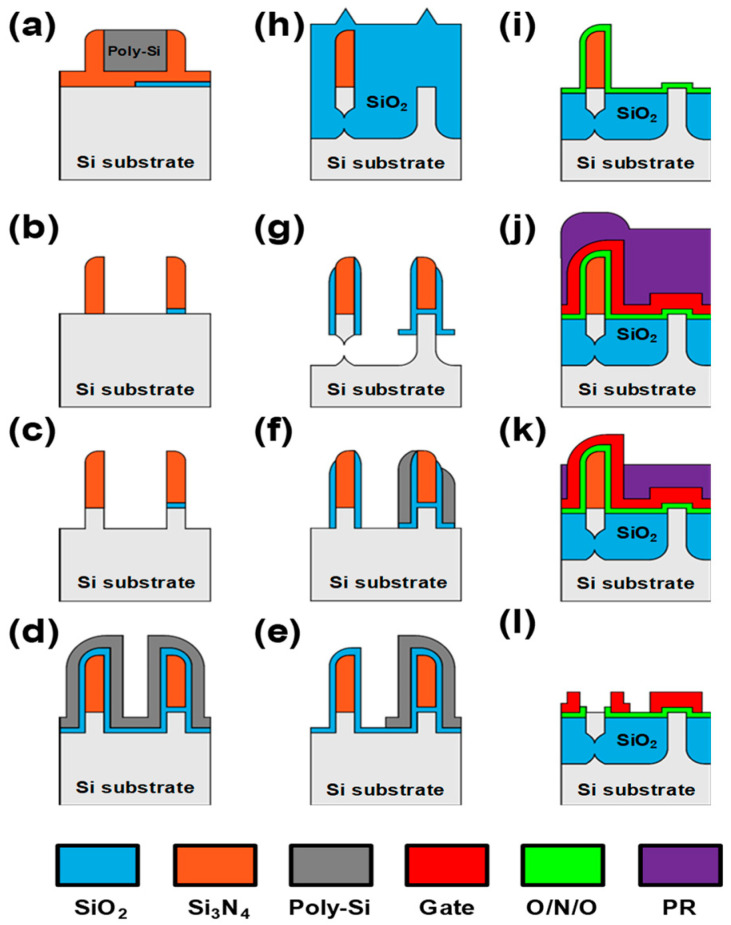
(**a**–**l**) Steps for integration of the fabricated S-G FET, n-type FET, p-type FET, and memory devices on the same wafer.

The S-G FET was fabricated on a 6-inch Si wafer using conventional CMOS technology. Figure 2a–h shows the fabrication steps for integrating the S-G FET, n-type FET, p-type FET, and memory devices. First, a sacrificial SiO_2_ layer is deposited on the wafer and patterned to obtain the single-gate FETs, and a nitride layer is then deposited. Subsequently, a 250 nm poly-Si layer is deposited and patterned to enable the formation of the Si_3_N_4_ spacers. Next, a 35 nm (fin width = 35 nm) Si_3_N_4_ layer is deposited and etched again, causing the Si_3_N_4_ spacers to be formed on the sidewalls of the patterned poly-Si layer (Figure 2a). The poly-Si layer is selectively removed using a solution of HNO_3_ and HF (Figure 2b), and the Si substrate is etched to a depth of 80 nm (fin height = 80 nm) with the Si_3_N_4_ spacers as a hard mask (Figure 2c). Then, a thin SiO_2_ film is again deposited as a barrier layer for SF_6_ dry etching, followed by the deposition of a poly-Si layer (Figure 2d). The poly-Si spacers are selectively removed using a mask in the regions of bulk-fin FETs, followed by anisotropic etching of SiO_2_ (Figure 2e). Thereafter, isotropic Si etching using SF_6_ gas is performed by controlling the etching thickness (Figure 2f). Si fins without the SiO_2_/poly-Si sidewalls are separated from the Si substrate, as shown on the left in Figure 2g. Notably, both ends of the separated fins are connected to the substrate, as shown on the right in Figure 2g. By wet-etching SiO_2_ in a buffered hydrogen fluoride solution, the Si_3_N_4_ spacers on the sacrificial SiO_2_ are removed. The gap between the Si fins is filled with SiO_2_ via a high-density plasma chemical vapor deposition process (Figure 2h). After the SiO_2_ is etched up to a certain thickness (until the top of the Si fins), boron and phosphorus ions are implanted for the field and channel doping of the n-type and p-type FETs, respectively. Then, the SiO_2_/Si_3_N_4_/SiO_2_ (2/4.2/9 nm) gate dielectric layer, which is called the O/N/O layer, is deposited for a memory function, and a SiO_2_ film (10 nm) is deposited as the gate dielectric layer of the conventional MOSFET, respectively. (Figure 2i). Then, an *n*^+^-doped poly-Si layer is deposited as a gate material. To form the independent split gates using the Si_3_N_4_ spacers, the wafer is coated with a diluted photoresist (PR); a thin PR layer is then formed only on the top of the Si_3_N_4_ spacers (Figure 2j). By etching the PR up to a specific thickness, only the *n*^+^-doped poly-Si on the Si_3_N_4_ spacers is exposed (Figure 2k). The exposed *n*^+^-doped poly-Si is then etched to form the independent split gates. The remaining PR is removed, followed by the patterning of the *n*^+^-doped poly-Si for the gates. Subsequently, the Si_3_N_4_ spacer is striped (Figure 2l). After boron and arsenic ions are implanted for the source/drain regions of the n-type and p-type FETs, respectively, rapid thermal annealing is conducted at a temperature of 1050 °C (5 s) to activate the ions. Then, an interlayer dielectric is deposited, and the contact holes and metal are patterned.

**Figure 3:**
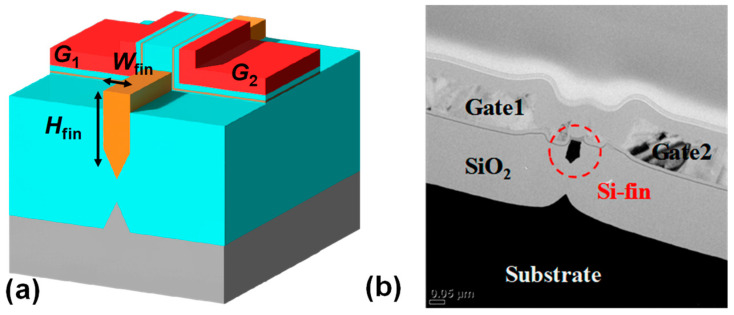
(**a**) 3D schematic view and (**b**) cross-sectional TEM image of the fabricated S-G FET.

Figure 3a,b show a 3D schematic view and a cross-sectional transmission electron microscopy (TEM) image, respectively, of the fabricated S-G FET with a floating fin body. The split gates (G1 and G2) are n+ doped poly-Si, and the thickness of the gate oxide (SiO_2_) is 9 nm for the conventional CMOSFET. The thicknesses of the tunneling layer, charge-trap layer, and blocking layer are 2 nm, 4.2 nm, and 9 nm, respectively, consisting of the SiO_2_/Si_3_N_4_/SiO_2_ stack to provide the nonvolatile memory for the homeostatic function. The width (*W*_fin_) and height (*H*_fin_) of the floating fin body are 35 nm and 80 nm, respectively. The split gates on both sides of the floating fin body can be used to modulate the threshold voltage (V_th_) of the S-G FET, thereby enabling neuron circuits to be controlled using the S-G FET. The doping concentrations of the n-type channel, p-type channel, source region, and drain region are 1 × 10^18^ cm^−3^, 1 × 10^18^ cm^−3^, 2 × 10^20^ cm^−3^, and 2 × 10^20^ cm^−3^, respectively.

## 3. Results and Discussion

### 3.1. Basic Device Characteristics

**Figure 4:**
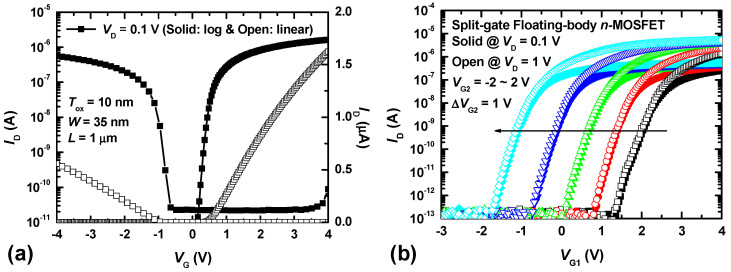
(**a**) The measured *I*_D_-*V*_G_ curves of the n-type FET and p-type FET, fabricated on the same wafer with the S-G FET. (**b**) The measured *I*_D_-*V*_G1_ curves of the S-G FET as a parameter of different *V*_G2_ from −2 V to 2 V, respectively. The solid and open symbols represent transfer curves at *V*_D_ of 0.1 V and 1 V, respectively.

Figure 4 shows the I_D_-V_G_ characteristics of CMOSs and S-G FET, respectively. The entire measurement process was conducted using the Keysight B1500A parameter analyzer with 10ms intervals. Figure 4a shows the measured I_D_–V_G_ curves of the n-type and p-type FETs fabricated on the same wafer. The gate width (W) and length (L) of the conventional CMOSs are 35 nm and 1 μm, respectively. The subthreshold swings (SS) of of conventional n-type MOSFET and p-type MOSFET are 70 mV/decade and 160 mV/decade at V_D_ = 0.1 V, respectively. The conventional CMOSs are used for the neuron circuits, self-controller (including a pulse generator for the program/erase operation of synaptic devices and the S-G FET), and connecting parts (current mirror and switch circuits) between the synaptic array and neuron circuit. Figure 4b shows the measured I_D_–V_G1_ curves of the S-G FET for V_G2_ values between −2 V and 2 V. The solid and hollow symbols represent the transfer curves at V_D_ values of 0.1 V and 1 V, respectively. For both sets of curves, the I_D_ values are almost identical at the same V_G1_ due to the low drain-induced barrier lowering of the fabricated S-G FET. As the gate voltage applied to G2 decreases from positive to negative, the potential in the channel region increases, and the electron energy barrier from the source region to the channel region increases as well. This results in an increase in the V_th_ of the S-G FET. Since the channel region between the split gates is fully depleted as 35 nm, the △V_th_ of the S-G FET is almost linearly related to △V_G2_ [20]. In previous studies, neuron circuits were designed as homeostatic circuits consisting of current mirrors and a single capacitor, to mimic the biological homeostatic function. However, since this involves storing the homeostatic data of neurons in capacitors to control the V_th_ of each neuron, a risk of data loss exists due to the volatile memory characteristics of the capacitors. Additionally, the large size of such capacitors (with a 1 pF capacitor occupying 100 μm² at a SiO_2_ thickness of 10 nm) causes the neuron circuit array to occupy a significant area. As described below, however, utilizing the nonvolatile memory characteristics of the charge-trap layer allows for implementing the homeostatic function of biological neurons at a high density as well as permanent storage of homeostatic data.

**Figure 5:**
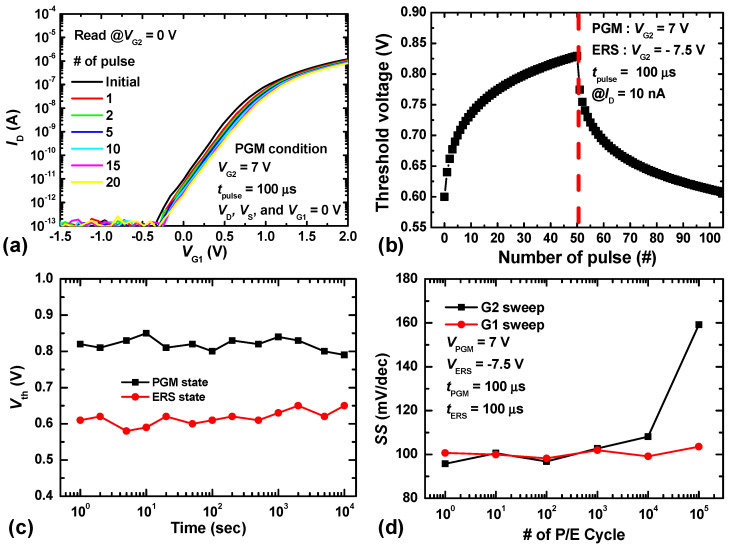
(**a**) *I*_D_-*V*_G1_ curves of the S-G FET as a function of the number of pulses applied to G2. (**b**) V_th_ variation in *I*_D_-*V*_G1_ curves of the S-G FET with the number of program/erase pulses applied to G2 at an *I*_D_ of 10 nA. (**c**) Retention characteristics **(**V_th_ change) of the S-G FET over time. (**d**) *SS* variation in the G1 and G2 DC sweep of the S-G FET with the number of program/erase cycles applied to G2.

When the S-G FET with the two independent gates (G1 and G2) is used in a neuron circuit, one gate (G1) reads input signals from a synaptic array, while the other gate (G2) modulates the V_th_ of the S-G FET through the amount of charge in the charge-trap layer via program/erase operations. The program/erase operations are executed in the tunneling layer through the Fowler–Nordheim (FN) tunneling mechanism when high positive/negative voltages are biased across the gate dielectric layers. Figure 5a shows the measured I_D_–V_G1_ curves of the S-G FET with the number of program pulses applied to G2. When a positive pulse (V_PGM_ of 7 V, t_PGM_ of 100 μs) is applied to G2 for the program operation, electrons accumulate in the charge-trap layer adjacent to G2; this has the same effect as applying a negative voltage to G2, resulting in an increase in the V_th_ of the S-G FET. By contrast, when a negative pulse (V_ERS_ of −7.5 V, t_ERS_ of 100 μs) is applied to G2 for the erase operation, the accumulated electrons are de-trapped, resulting in a decrease in the V_th_ of the S-G FET. Figure 5b shows the measured V_th_ changes in I_D_–V_G1_ curves of the S-G FET with the number of program/erase pulses applied to G2; V_th_ is measured at an I_D_ of 10 nA. As the number of program pulses applied to G2 increases, V_th_ increases from 0.6 V to 0.83 V at 50 pulses. Conversely, when the erase pulses are applied to G2, V_th_ gradually decreases and returns to its initial value of 0.6 V when more than 50 pulses are applied. V_th_s in program and erase states were measured at room temperature with the terminals open. As a result, The V_th_s of the S-G FET in both states are maintained well over 10^4^ s (< V_th_ changes of 10%), as shown in Figure 5c. The V_th_ values of the S-G FET in both the program and erase states are maintained for well over 10^4^ s (V_th_ variation < 10%), as shown in Figure 5c; the nonvolatile memory function enables the optimized V_th_ of the S-G FET to be maintained. If the S-G FET is used to determine the threshold of one neuron circuit during the learning process of an SNN, the threshold of all other neuron circuits can be optimized through the PGM/ERS operation. Moreover, G1 and G2 being independent gates allows for the thresholds of all neuron circuits to be updated selectively. No change in the SS is observed until 10^4^ program/erase cycles have been executed. However, beyond 10^5^ cycles, the tunneling layer on G2 becomes degraded, resulting in an increase in the SS corresponding to the V_G2_ of the S-G FET. Notably, since the program/erase pulses are applied to G2, the gate dielectric of G1 does not degrade, even after more than 10^5^ program/erase cycles, as seen in Figure 5d. In addition, |7 V| program/erase pulses are applied to G2 of the S-G FET to modulate the threshold value of the neuron circuit only during the learning process. After learning, only small signal (synaptic signals, membrane potential) are applied to G1 of the S-G FET for the classification and pattern recognition. Therefore, when implementing biological homeostasis functions using nonvolatile memory characteristics, signals transmitted from the synaptic array can be reliably read.

### 3.2. A Neuron Circuit Using the S-G FET

**Figure 6:**
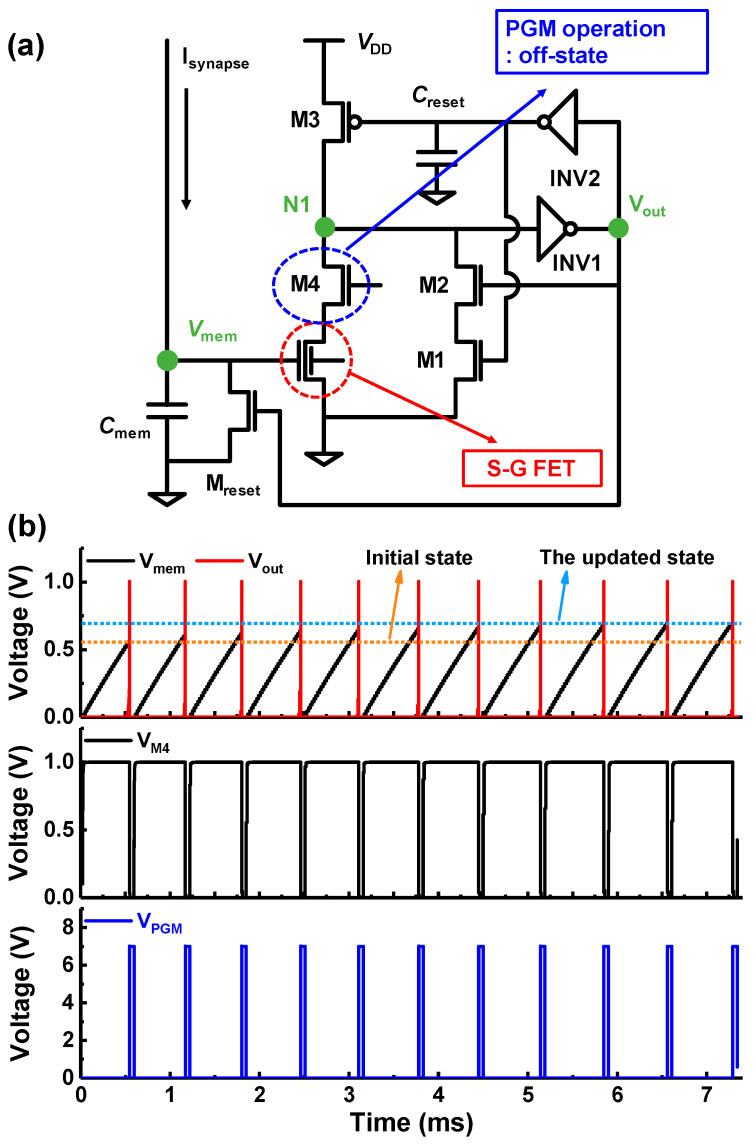
(**a**) Schematic of IF neuron circuit integrated with S-G FET. (**b**) Simulated operational characteristics demonstrating the increase in the neuron circuit threshold during the learning process.

Figure 6a shows a schematic of an integrate-and-fire (IF) neuron circuit integrated with the S-G FET. The S-G FET is designed using a Sentaurus TCAD simulation, and the modulation of the V_th_ in the charge trap layer (Si_3_N_4_) of the S-G FET was performed through the program and erased operations via the Fowler-Nordheim (FN) tunneling model. When determining carrier mobility, the velocity saturation model, doping dependence model, and Lombardi surface mobility model were used. The old Slotboom model was used to calculate bandgap narrowing due to doping. The simulation of the neuron circuit with the S-G FET was conducted using the mixed mode provided in the Sentaurus TCAD simulation. The parameters of the CMOS and capacitors are as follows: L = 0.5 μm, W = 0.1 μm, C_mem_ = 0.5 pF, C_reset_ = 0.05 pF. The supply voltage (V_DD_) is 1.0 V. C_mem_ is used for the integrate function, while the S-G FET compares the membrane potential (V_mem_) with V_th_. When V_mem_ exceeds the V_th_ of the S-G FET due to the charge stored in C_mem_, the S-G FET triggers the fire function, and the state of Node 1 (N1) changes from high to low. Then, the output node (V_out_) of INV1 changes from a low to a high state, and the charges accumulated on C_mem_ are drained through M_reset_. Finally, the neuron circuit resets to the initial state via a feedback signal. At this point, M1 and M2 temporarily enhance the pull-down operation, whereby the S-G FET is activated and lowers the voltage at N1. M4 is intended to prevent the drain current from flowing through the S-G FET when V_PGM_ (positive voltage) is applied to G2 for the program operation. Figure 6b demonstrates the IF operation of the neuron circuit using the S-G FET during the learning process. Through this operation, the output signal is transmitted to the expended pulse generator and voltage shifter, where it can be modulated to the desired pulses for the program operation. When a program pulse (V_PGM_ of 7 V, t_PGM_ of 100 μs) is applied to G2, 0 V is simultaneously applied to the M4 gate to perform the program operation through FN tunneling. The neuron circuit performs the IF operation at a higher V_mem_ from 0.56 V, as shown in Figure 6b. Through the program operation of the S-G FET, V_th_s of the neuron circuit are increased. Each of the V_th_s of the neuron circuit after the program operation was 0.558 V, 0.60 V, 0.618 V, 0.637 V, 0.66 V, 0.675 V, and 0.71 V. Consequently, the firing rate of the neuron circuit also linearly decreases as 1902 Hz, 1754 Hz, 1694 Hz, 1639 Hz, 1587 Hz, 1538 Hz, and 1449 Hz. Raising the threshold of frequently firing neuron circuits allows other neurons a greater chance of firing.

**Figure 7:**
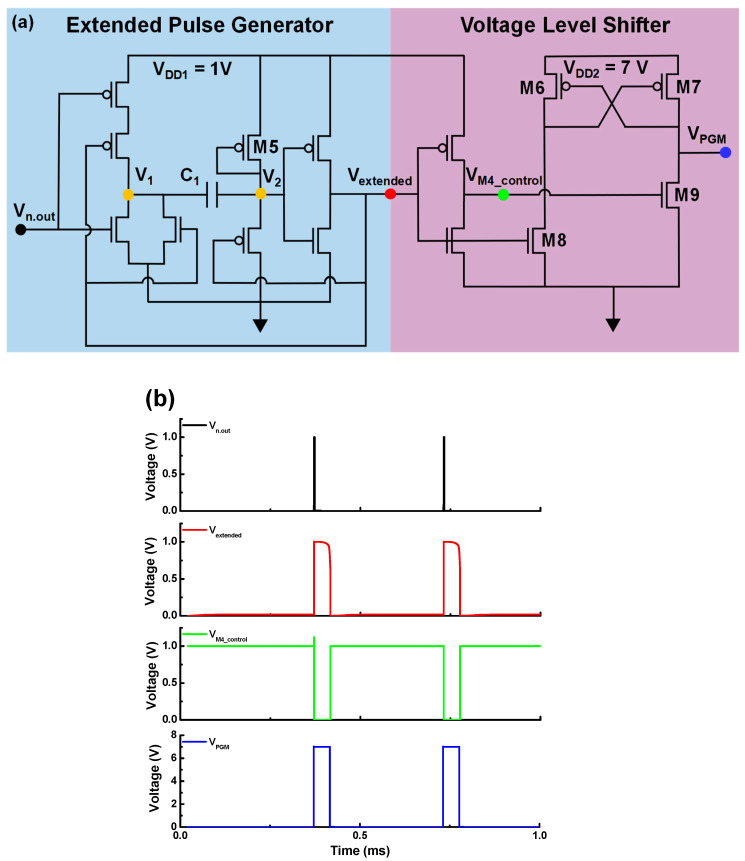
(**a**) Basic configuration circuit including extended pulse generation and voltage level shifter for program/erase operations. (**b**) Simulated *V*–*t* plots for modulation of *V*_PGM_ pulse from the output pulse of the neuron circuit using the basic configuration circuit.

An output spike of 1 V and 2 μs generated by the neuron circuit is insufficient for the program/erase operations required to inject charge into the charge-trap layer for implementing homeostatic functions. Hence, to generate the required program/erase pulses, an extended pulse generator and a voltage level shifter are designed (Figure 7a) [57]. The extended pulse generator consists of a NOR gate and buffer invertors to extend the width of the pulse, while the voltage level shifter comprises an inverter and differential amplifier to raise the output signal of the neuron to the *V*_PGM_ level. In Figure 7a, the parameters of the CMOS, excluding M5, M6, and M7, are as follows: L = 0.5 μm, W = 0.1 μm, C_1_ = 1 pF, *V*_DD1_ = 1 V, and *V*_DD2_ = 7 V. M5 is designed with L = 2 μm and W = 0.1 μm to slowly pull up the *V*_2_ node; the width of the extended pulse is determined by M5 and the capacitor C_1_. Standard CMOS devices are unsuitable for handling high supply voltage about 7 V, in the case of M6 to M9 for high voltage, the simulation was conducted by setting it as a device with lightly doped drain (LDD) which is doping concentration of 1 × 10^19^ cm^− 3^ [58,59]. M6 and M7 are designed with L = 5 um and W = 0.1 um to ensure an output voltage of 0 V in the off state, even when the applied *V*_DD2_ has a high value of 7 V or more. A high supply voltage is applied to the gates of M6 and M9, generating 7V, which can cause damage or degradation to the gate oxide. To ensure the stable operation of the circuit, it is necessary to improve the quality and increase the thickness of the CMOS gate oxide through fabrication.

When the output spike is transmitted to the V_n.out_ node, the NOR gate lowers V_1_ from 1 V to 0 V. Since V_2_ is coupled to V_1_ by C_1_, V_2_ is also set to the low state. Consequently, V_extended_ is raised to the high state by the inverter. Even though V_n.out_ changes from 1 V to 0 V after 2 μs (t_spike_ = 2 μs), V_1_ remains 0 V because V_extended_, another input voltage to the NOR gate, is 1 V. However, when V_2_ is 0 V, M5 is activated, which causes V_2_ to increase gradually from 0 V to 1 V. As shown in Figure 7b, when V_2_ is close to 1 V after 50 μs, V_extended_ decreases from 1 V to 0 V. Since all the input voltages and the output voltage of the NOR gate become 0 V, both V_1_ and V_2_ return to the initial value of 1 V. Thus, the extended pulse generator allows for extending the output spike of the neuron circuit to the time required for the program operation, and the extension duration is determined by the capacitance of C_1_ and the current at M5. Next, the process of modulating the amplitude of the extended pulse from 1 V to 7 V is described. The output voltage of the extended pulse generator is transmitted to the input node of the voltage level shifter and the gate of M8. When V_extended_ is raised, M8 is activated, and the inverter deactivates M9. Thus, M7 is activated, and a V_DD2_ of 7 V is applied as the output voltage of the voltage level shifter, which is used as the V_PGM_ for the program operation of the synaptic devices and the S-G FET. Conversely, lowering V_extended_ deactivates M8, causes the inverter to activate M9, and returns all nodes to their initial condition, as shown in Figure 7b.

### 3.3. Pattern Recognition in SNN with Homeostasis Function

**Figure 8:**
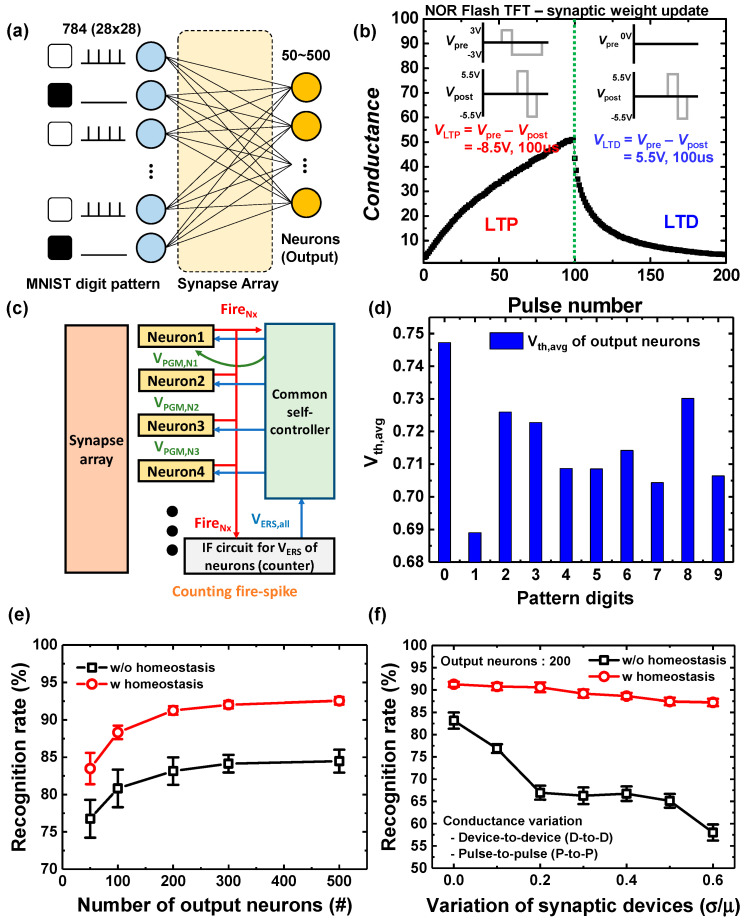
(**a**) A diagram of a fully connected neural network with a synapse array and neurons. (**b**) Synaptic weight updates (LTP/LTD) and operation scheme of a TFT memory device [60] (**c**) A block diagram of the neuromorphic system consisting of a synapse array, neurons, and a common controller. (**d**) The optimized threshold voltages of output neurons with the input patterns. (**e**) Comparison of the pattern recognition accuracy in STDP based SNN system with/without homeostasis functionality. (**f**) The degradation of the recognition rate with increasing conductance variation (σ/μ) of the synaptic devices.

To verify the performance of a neural network based on the S-G FET, online unsupervised learning and classification were performed on in SNNs using MNIST data set with a Python simulator as shown in Figure 8a. The MNIST dataset is represented by 28 × 28 pixels, and 60,000 training and 10,000 test images of MNIST dataset are used for training and verification. The simulated SNN consists of a 784-input neuron layer and a 50–500 output neuron layer. To analyze the change in recognition rate according to the number of output neurons, the output neurons were set from 50 to 500. The synapse array for training the SNN used TFT (Thin Film Transistor) type NOR flash memory, and the memory characteristics for training used a simplified STDP, as shown in Figure 8b [60]. When V_pre_ and V_post_ are applied to the gate and source of each TFT synaptic device, respectively, program (LTD) and erase operations (LTP) in the charge storage layer are performed due to the potential difference (V_pre_–V_post_) between the gate and source [60]. Figure 8c shows a block diagram of the proposed system, consisting of a synapse array, neuron circuits, and a common controller (including switch circuits, the extended pulse generator, and the voltage level shifter). To ensure that other neurons have the opportunity to fire, the thresholds of frequently fired neurons were increased by applying V_PGM_SGFET_ whenever a neuron fired and transmitted an output signal. To accelerate the learning, the thresholds of all neuron circuits were reduced by periodically applying V_ERS_SGFET_ to all the neuron circuits. As described earlier, this operation can imitate the homeostasis functionality for controlling the firing rate of neuron circuits. The initial voltage threshold of each neuron in the proposed neural network is 0.7 V, and Figure 8d,e show the optimized V_th_ of the output neurons and the recognition rate of the proposed system, respectively, for a given MNIST dataset. The recognition rate is an average of 10 runs. The type of MNIST digits can be distinguished better by increasing the number of output neurons in a neural computing system, which leads to highly accurate pattern recognition [20]. The maximum accuracy of the SNN based on the proposed homeostasis functionality reached 91.84% with 200 output neurons. The proposed SNN achieved about an 8% higher recognition rate than an SNN without the homeostasis function. In addition, although the conductance fluctuation of synaptic devices is large (σ/μ > 0.5), as shown in Figure 8f, the degradation of the recognition rate was observed to be low (~3%) in the proposed system due to the homeostasis function. However, the recognition rate without the homeostasis functionality was severely compromised as the fluctuation of the synaptic devices increased.

## 4. Conclusions

A S-G FET was designed and fabricated in this study to control the threshold of neuron circuits for spiking neural networks. The V_th_ variation of the S-G FET was verified experimentally by applying a V_PGM_ of 7 V and V_ERS_ of −7.5 V to the G2 gate. Even after more than 10^5^ program/erase cycles, the gate dielectrics of G1 did not degrade; thus, no change in the SS of G1 was observed. In neuron circuits with the homeostatic function, the recognition rate was improved by 8% by increasing the firing threshold of the circuits. Furthermore, the nonvolatile memory functionality of the charge storage layer in the S-G FET could maintain the thresholds (for over 10^4^ s) of the neuron circuits optimized during the SNN’s learning process. The proposed homeostatic neuron functionality yielded a high classification accuracy for the MNIST dataset, despite large fluctuations in the synaptic devices in the two-layer SNN.

## Data Availability

The original contributions presented in the study are included in the article, further inquiries can be directed to the corresponding author/s.

## References

[1] Yu S. (2018). Neuro-inspired computing with emerging nonvolatile memories. Proc. IEEE.

[2] Masquelier T., Thorpe S.J. (2007). Unsupervised learning of visual features through spike timing dependent plasticity. PLoS Comput. Biol..

[3] Wu X., Saxena V., Zhu K. (2015). Homogeneous Spiking Neuromorphic System for Real-World Pattern Recognition. IEEE J. Emerg. Sel. Top. Circuits Syst..

[4] Bichler O., Querlioz D., Thorpe S.J., Bourgoin J.-P., Gamrat C. Unsupervised features extraction from asynchronous silicon retina through Spike-Timing-Dependent Plasticity. Proceedings of the 2011 International Joint Conference on Neural Networks.

[5] Qiao N., Mostafa H., Corradi F., Osswald M., Stefanini F., Sumislawska D., Indiveri G. (2018). A reconfigurable on-line learning spiking neuromorphic processor comprising 256 neurons and 128K synapses. Front. Neurosci..

[6] Ferre P., Mamalet F., Thorpe S.J. (2018). Unsupervised Feature Learning with Winner-Takes-All Based STDP. Front. Comput. Neurosci..

[7] Diehl P.U., Cook M. (2015). Unsupervised learning of digit recognition using spike-timing-dependent plasticity. Front. Comput. Neurosci..

[8] Querlioz D., Bichler O., Dollfus P., Gamrat C. (2013). Immunity to device variations in a spiking neural network with memristive nanodevices. IEEE Trans. Nanotechnol..

[9] Park J., Kim S., Song M.S., Youn S., Kim K., Kim H. (2024). Implementation of convolutional neural networks in memristor crossbar arrays with binary activation and weight quantization. ACS Appl. Mater. Interfaces.

[10] Wang Y., Yin L., Huang W., Li Y., Huang S., Zhu Y., Yang D., Pi X. (2021). Optoelectronic synaptic devices for neuromorphic computing. Adv. Intell. Syst..

[11] Florini D., Gandolfi D., Mapelli J., Benatti L., Pavan P., Puglisi F.M. (2024). A Hybrid CMOS-Memristor Spiking Neural Network Supporting Multiple Learning Rules. IEEE Trans. Neural Netw. Learn. Syst..

[12] Milo V., Pedretti G., Carboni R., Calderoni A., Ramaswamy N., Ambrogio S., Ielmini D. Demonstration of hybrid CMOS/RRAM neural networks with spike time/rate-dependent plasticity. Proceedings of the 2016 IEEE International Electron Devices Meeting (IEDM).

[13] Cooper L., Bear M. (2012). The BCM theory of synapse modification at 30: Interaction of theory with experiment. Nat. Rev. Neurosci..

[14] Ahmed T., Walia S., Mayes E.L.H., Ramanathan R., Bansal V., Bhaskaran M., Sriram S., Kavehei O. (2019). Time and rate dependent synaptic learning in neuro-mimicking resistive memories. Sci. Rep..

[15] Fernandes D., Carvalho A.L. (2016). Mechanisms of homeostatic plasticity in the excitatory synapse. J. Neurochem..

[16] Wang G., Gilbert J., Man H.-Y. (2012). AMPA Receptor Trafficking in Homeostatic Synaptic Plasticity: Functional Molecules and Signaling Cascades. Neural Plast..

[17] Tien N.W., Kerschensteiner D. (2018). Homeostatic plasticity in neural development. Neural Dev..

[18] Chowdhury D., Hell J.W. (2018). Homeostatic synaptic scaling: Molecular regulators of synaptic AMPA-type glutamate receptors. F1000Research.

[19] Kim K., Song M.S., Hwang H., Hwang S., Kim H. (2024). A comprehensive review of advanced trends: From artificial synapses to neuromorphic systems with consideration of non-ideal effects. Front. Neurosci..

[20] Cho S. (2022). Volatile and nonvolatile memory devices for neuromorphic and processing-in-memory applications. J. Semicond. Technol. Sci..

[21] Lee G.H., Song M.S., Kim S., Yim J., Hwang S., Yu J., Kwon D., Kim H. (2022). Ferroelectric field-effect transistors for binary neural network with 3-D NAND architecture. IEEE Trans. Electron Devices.

[22] Li Z., Meng J., Yu J., Liu Y., Wang T., Liu P., Chen S., Zhu H., Sun Q., Zhang D.W. (2023). CMOS compatible low power consumption ferroelectric synapse for neuromorphic computing. IEEE Electron Device Lett..

[23] Kumar A., Krishnaiah M., Park J., Mishra D., Dash B., Jo H.-B., Lee G., Youn S., Kim H., Jin S.H. (2024). Multibit, lead-free Cs_2_SnI_6_ resistive random access memory with self-compliance for improved accuracy in binary neural network application. Adv. Funct. Mater..

[24] Kim J.P., Kim S.K., Park S., Kuk S.-H., Kim T., Kim B.H., Ahn S.-H., Cho Y.-H., Jeong Y., Choi S.-Y. (2023). Dielectric-engineered high-speed, low-power, highly reliable charge trap flash-based synaptic device for neuromorphic computing beyond inference. Nano Lett..

[25] Youn S., Lee J., Kim S., Park J., Kim K., Kim H. (2024). Programmable threshold logic implementations in a memristor crossbar array. Nano Lett..

[26] Han X., Zhang J., Zeng T., Zhao X., Li J., Sun H., Cao Y., Tong Y., Tang Q., Liu Y. (2023). Super-flexible, transparent synaptic transistors based on pullulan for neuromorphic electronics. IEEE Electron Device Lett..

[27] Kim S., Park J., Kim T.-H., Hong K., Hwang Y., Park B.G., Kim H. (2022). 4-bit Multilevel Operation in Overshoot Suppressed Al_2_O_3_/TiO_x_ Resistive Random-Access Memory Crossbar Array. Adv. Intell. Syst..

[28] Hwang S., Yu J., Song M.S., Hwang H., Kim H. (2023). Memcapacitor crossbar array with charge trap NAND flash structure for neuromorphic computing. Adv. Sci..

[29] Kim T.-H., Kim S., Hong K., Park J., Youn S., Lee J.-H., Park B.-G., Kim H. (2022). Effect of program error in memristive neural network with weight quantization. IEEE Trans. Electron Devices.

[30] Werner T., Vianello E., Bichler O., Grossi A., Nowak E., Nodin J.-F., Yvert B., DeSalvo B., Perniola L. Experimental demonstration of short and long term synaptic plasticity using OxRAM multi k-bit arrays for reliable detection in highly noisy input data. Proceedings of the IEEE International Electron Devices Meeting (IEDM).

[31] Wang Z.Q., Xu H.Y., Li X.H., Yu H., Liu Y.C., Zhu X.J. (2012). Synaptic learning and memory functions achieved using oxygen ion migration/diffusion in an amorphous InGaZnO memristor. Adv. Funct. Mater..

[32] Oh S., Huang Z., Shi Y., Kuzum D. (2019). The impact of resistance drift of phase change memory (PCM) synaptic devices on artificial neural network performance. IEEE Electron Device Lett..

[33] Bianchi S., Munoz-Martin I., Hashemkhani S., Pedretti G., Ielmini D. A bio-inspired recurrent neural network with self-adaptive neurons and PCM synapses for solving reinforcement learning tasks. Proceedings of the IEEE International Symposium on Circuits and Systems (ISCAS).

[34] Kang M., Park J. Peripheral circuit optimization with precharge technique of spin transfer torque MRAM synapse array. Proceedings of the International Technical Conference on Circuits/Systems, Computers and Communications (ITC-CSCC).

[35] Feng Y., Huang P., Zhao Y., Shan Y., Zhang Y., Zhou Z., Liu L., Liu X., Kang J. (2021). Improvement of state stability in multi-level resistive random-access memory (RRAM) array for neuromorphic computing. IEEE Electron Device Lett..

[36] Li Y., Ang K.-W. (2021). Hardware implementation of neuromorphic computing using large-scale memristor crossbar arrays. Adv. Intell. Syst..

[37] Jeon K., Kim J., Ryu J.J., Yoo S.-J., Song C., Yang M.K., Jeong D.S., Kim G.H. (2021). Self-rectifying resistive memory in passive crossbar arrays. Nat. Commun..

[38] Hsieh E., Zheng X., Le B., Shih Y., Radway R., Nelson M., Mitra S., Wong S. (2021). Four-bits-per-memory one-transistor-and-eight-resistive-random-access-memory (1T8R) array. IEEE Electron Device Lett..

[39] Rajendran B., Alibart F. (2016). Neuromorphic Computing Based on Emerging Memory Technologies. IEEE J. Emerg. Sel. Top. Circuits Syst..

[40] Yang J.Q., Zhou Y., Han S.T. (2021). Functional Applications of Future Data Storage Devices. Adv. Electron Mater..

[41] Li S., Lyu H., Li J., He Y., Gao X., Wan Q., Shi Y., Pan L. (2021). Multiterminal ionic synaptic transistor with artificial blink reflex function. IEEE Electron Device Lett..

[42] Lee S.-T., Lim S., Choi N.Y., Bae J.-H., Kwon D., Park B.-G., Lee J.-H. (2019). Operation scheme of multi-layer neural networks using nand flash memory as high-density synaptic devices. IEEE J. Electron Devices Soc..

[43] Park Y.J., Kwon H.T., Kim B., Lee W.J., Wee D.H., Choi H.-S., Park B.-G., Lee J.-H., Kim Y. (2018). 3-D stacked synapse array based on charge-trap flash memory for implementation of deep neural networks. IEEE Trans. Electron Devices.

[44] Woo S.Y., Choi K.-B., Kim J., Kang W.-M., Kim C.-H., Seo Y.-T., Bae J.-H., Park B.-G., Lee J.-H. (2020). Implementation of homeostasis functionality in neuron circuit using double-gate device for spiking neural network. Solid-State Electron..

[45] Wu H., Yao P., Gao B., Wu W., Zhang Q., Zhang W., Deng N., Wu D., Wong H.-S.P., Yu S. Device and circuit optimization of RRAM for neuromorphic computing. Proceedings of the 2017 IEEE International Electron Devices Meeting (IEDM).

[46] Lim S., Bae J.-H., Eum J.-H., Lee S., Kim C.-H., Kwon D., Park B.-G., Lee J.-H. (2018). Adaptive learning rule for hardware-based deep neural networks using electronic synapse devices. Neural Comput. Appl..

[47] Kwon D., Lim S., Bae J.-H., Lee S.-T., Kim H., Kim C.-H., Park B.-G., Lee J.-H. (2019). Adaptive Weight Quantization Method for Nonlinear Synaptic Devices. IEEE Trans. Electron Devices.

[48] Woo S.Y., Choi K.-B., Lim S., Lee S.-T., Kim C.-H., Kang W.-M., Kwon D., Bae J.-H., Park B.-G., Lee J.-H. (2019). Synaptic device using a floating fin-body MOSFET with memory functionality for neural network. Solid-State Electron..

[49] Bartolozzi C., Nikolayeva O., Indiveri G. Implementing homeostatic plasticity in VLSI networks of spiking neurons. Proceedings of the 15th IEEE International Conference on Electronics, Circuits and Systems.

[50] Bartolozzi C., Indiveri G. (2009). Global scaling of synaptic efficacy: Homeostasis in silicon synapses. Neurocomputing.

[51] Rovere G., Ning Q., Bartolozzi C., Indiveri G. Ultra Low Leakage Synaptic Scaling Circuits for Implementing Homeostatic Plasticity in Neuromorphic Architectures. Proceedings of the 2014 IEEE International Symposium on Circuits and Systems (ISCAS).

[52] Qiao N., Indiveri G., Bartolozzi C. Automatic gain control of ultra-low leakage synaptic scaling homeostatic plasticity circuits. Proceedings of the 2016 IEEE Biomedical Circuits and Systems Conference (BioCAS).

[53] Shi X., Zeng Z., Yang L., Huang Y. (2018). Memristor-Based Circuit Design for Neuron with Homeostatic Plasticity. IEEE Trans. Emerg. Top. Comput. Intell..

[54] Zjajo A. Dynamic Homeostatic Regulation in Energy-Efficient Time-Locked Neuromorphic Systems. Proceedings of the 2020 IEEE 20th International Conference on Bioinformatics and Bioengineering (BIBE).

[55] Zhao Z., Qu L., Wang L., Deng Q., Li N., Kang Z., Guo S., Xu W. (2020). A Memristor-Based Spiking Neural Network with High Scalability and Learning Efficiency. IEEE Trans. Circuits Syst. II Express Briefs.

[56] Johnson A.P., Liu J., Millard A.G., Karim S., Tyrrell A.M., Harkin J., Timmis J., McDaid L.J., Halliday D.M. (2018). Homeostatic Fault Tolerance in Spiking Neural Networks: A Dynamic Hardware Perspective. IEEE Trans. Circuits Syst. I Regul. Pap..

[57] Kang W.-M., Kim C.-H., Lee S., Woo S.Y., Bae J.-H., Park B.-G., Lee J.-H. A spiking neural network with a global self-controller for unsupervised learning based on spike-timing-dependent plasticity using flash memory synaptic devices. Proceedings of the 2019 International Joint Conference on Neural Networks (IJCNN).

[58] Goda A. (2021). Recent Progress on 3D NAND Flash Technologies. Electronics.

[59] Woo S.Y., Kang W.M., Seo Y.T., Lee S., Kwon D., Oh S., Bae J.-H., Lee J.-H. (2022). Demonstration of integrate-and-fire neuron circuit for spiking neural networks. Solid-State Electron..

[60] Kim C.-H., Lee S., Woo S.Y., Kang W.-M., Lim S., Bae J.-H., Kim J., Lee J.-H. (2018). Demonstration of Unsupervised Learning with Spike-Timing-Dependent Plasticity Using a TFT-Type NOR Flash Memory Array. IEEE Trans. Electron Devices.

